# Efficacy and Safety of Molnupiravir in Mild COVID-19 Patients in India

**DOI:** 10.7759/cureus.31508

**Published:** 2022-11-14

**Authors:** Shubhadeep Sinha, Kumarasamy N, Vasanth Kumar Suram, Sreenivasa S Chary, Sunil Naik, Veer Bahadur Singh, Manish K Jain, Chandra P Suthar, Swapnav Borthakur, Vinayak Sawardekar, Noushadali Sk, Naveen Reddy, Leela Talluri, Pankaj Thakur, Mohan Reddy, Muralidhar Panapakam, Ramya Vattipalli

**Affiliations:** 1 Clinical Development and Medical Affairs, Hetero, Hyderabad, IND; 2 Infectious Diseases Medical Centre, Voluntary Health Services, Chennai, IND; 3 Department of Medicine, Gandhi Hospital, Hyderabad, IND; 4 General Medicine, Rajiv Gandhi Institute of Medical Sciences, Srikakulam, IND; 5 General Medicine, Jawahar Lal Nehru Medical College, Bikaner, IND; 6 Pulmonology, Maharaja Agrasen Superspeciality Hospital, Jaipur, IND; 7 Medicine, Dana Shivam Heart & Superspeciality Hospital, Jaipur, IND; 8 Internal Medicine, Down Town Hospital, Guwahati, IND; 9 General Medicine, St. George’s Hospital, Mumbai, IND; 10 General Medicine, A. C. Subba Reddy Government Medical College, Nellore, IND; 11 Internal Medicine, AIG Hospitals, Hyderabad, IND; 12 Clinical Data Management, Hetero, Hyderabad, IND

**Keywords:** efficacy and safety, oral antiviral drug, phase 3 study, molnupiravir, sars-cov-2 infection

## Abstract

Background

At the peak of the coronavirus disease 2019 (COVID-19) pandemic, the need for an orally administered agent to prevent the progression of acute respiratory syndrome coronavirus 2 (SARS-CoV-2) infection became increasingly evident, which was the impetus behind our investigations with molnupiravir. Molnupiravir has been shown to be effective in preventing hospitalizations and/or clinical complications in patients with mild-to-moderate COVID-19. In this study, we evaluate the efficacy and safety of molnupiravir in Indian patients with mild SARS-CoV-2 infection and at least one risk factor for disease progression (CTRI/2021/05/033739).

Methodology

This was a phase III, multicenter, randomized, open-label, controlled study conducted in Indian adults aged 18-60 years with mild SARS-CoV-2, reverse transcription polymerase chain reaction (RT-PCR)-positive within 48 hours of enrollment in the study, and within five days of first symptom onset. Enrolled patients were randomized to treatment arms in a 1:1 ratio to receive molnupiravir or placebo in addition to the standard of care (SoC) for SARS-CoV-2 infection. The SoC was in compliance with Government of India guidelines that were in force at the time. The primary endpoint was the rate of hospitalization up to day 14. Safety endpoints included incidence of adverse events (AEs).

Results

Eligible patients were randomized in a 1:1 ratio to receive molnupiravir in addition to SoC treatment (n = 608) or SoC alone (n = 610). In the molnupiravir group, nine (1.48%) patients required hospitalization versus 26 (4.26%) patients in the control group (risk difference = -2.78%; 95% CI = -4.65, -0.90; p = 0.0053). Overall, 45 (3.70%) patients reported 47 AEs during the study, most of which were mild and resolved completely. The molnupiravir group reported 30 AEs compared to 17 AEs in the control group. Headache and nausea were the two most commonly reported AEs.

Conclusions

The molnupiravir arm showed a lower rate of hospitalization and a shorter time for the improvement of clinical symptoms coupled with early RT-PCR negativity. Molnupiravir was well tolerated, and AEs were mild and rare. The addition of molnupiravir to standard therapy has the potential to prevent the progression of mild COVID-19 disease to the severe form.

## Introduction

The limited and evolving understanding of severe acute respiratory syndrome coronavirus 2 (SARS-CoV-2) infection, pathogenesis, clinical symptomatology, and disease progression continues to pose a challenge in the discovery and development of effective antiviral therapies. Though some therapies such as remdesivir have been approved for use in coronavirus disease 2019 (COVID-19) by the United States Food and Drug Administration (USFDA) [1], numerous immunotherapies (i.e., tocilizumab, sotrovimab, casirivimab plus imdevimab, bamlanivimab plus etesevimab) initially received emergency use authorization (EUA) from the FDA [2], which were later revoked due to increasing concerns regarding their effectiveness against the omicron variant [3]. Recently, Bebtelovimab, with proven activity against the omicron variant, received EUA for use in patients with mild COVID-19 [4].

Oral medications that can be utilized much earlier in this infection and can be easily administered such as (PF-07321332/Ritonavir (Pfizer), and molnupiravir (MK-4482/EIDD-2801), a prodrug of ribonucleoside analog β-D-N4-hydroxycytidine, are currently approved under EUA and are perhaps the most advanced drug candidates in this category [5].

Published data from clinical trials have shown that molnupiravir is safe, effective, and well tolerated with no adverse effects or toxicity when administered to treat COVID-19 [6,7]. Oral administration makes molnupiravir suitable and convenient for outpatient administration, thus preventing mild COVID-19 patients from progressing to moderate/severe disease. During the first and second waves of the pandemic in India, treatment of moderate/severe COVID-19, especially in the intensive care unit (ICU), was a major challenge, making an effective and safe oral antiviral drug the need of the hour. Molnupiravir received its first global EUA in the United Kingdom based on interim results of the MOVe-OUT phase 3 clinical trial conducted by Merck Sharp & Dohme (MSD) and Ridgeback Biotherapeutics [8]. Based on the final results of MOVe-OUT trials, molnupiravir received a EUA from the USFDA for mild-to-moderate COVID-19 disease [9]. In line with global clinical trials, two separate phase 3 clinical trials, one in mild COVID-19 and another in moderate COVID-19 patients, were conducted in India. The results of the phase 3 study in mild COVID-19 patients are presented in this paper.

## Materials and methods

Study design and participants

This randomized, multicenter, open-label, comparative, parallel-group study was conducted at 23 multispecialty hospitals across India between May 2021 to August 2021 (CTRI /2021/05/033739). Male and female participants aged ≥18 to ≤60 years with mild COVID-19 disease (mild symptoms and uncomplicated upper respiratory tract infection without any evidence of breathlessness) who were found to be reverse transcriptase-polymerase chain reaction (RT-PCR) positive within 48 hours of enrollment and within five days of the first symptom onset were included. Female patients required a negative pregnancy test at enrolment and were counseled to avoid pregnancy during the study and for at least 28 days thereafter. Participants were excluded if they had moderate-to-severe COVID-19 (SpO_2_ ≤93% on room air or respiratory rate (RR) ≥24 breaths per minute, with or without pneumonia) [10], severe liver disease, active hepatitis C, B, or HIV, acute pancreatitis, or a history of chronic pancreatitis, severe renal impairment, or having received continuous renal replacement therapy.

This study was conducted in compliance with the Declaration of Helsinki (2013), Good Clinical Practice (ICH-GCP E6), and local regulatory guidelines. The study began with prior approval of the institutional ethics committee at each site, and all patients gave their written informed consent prior to enrolment in the study. In the case of illiterate patients, the impartial witness was present and signed informed consent.

Randomization

Patients meeting the screening criteria were randomized as per a central randomization scheme generated based on a random permuted block design with a block size of four and a 1:1 ratio between treatment groups using the statistical analysis software SAS® version 9.4 (SAS Institute Inc., NC, USA).

Study interventions

Molnupiravir group participants received molnupiravir 800 mg of (4 × 200 mg capsules, administered orally every 12 hours for five days) plus standard conventional therapy while the control group patients received standard of care (SoC) alone. The SoC treatment was as per the Clinical Guidance for Management of Adult COVID-19 Patients, dated April 22, 2021 [11], which included hydration, antipyretics, antitussive, multivitamins, ivermectin (200 µg/kg once a day for three days), or hydroxychloroquine (400 mg BD for one day f/b 400 mg OD for four days). Inhalational budesonide (800 µg BD for five days) was allowed if the symptoms (fever and/or cough) persisted beyond five days of disease onset.

Procedures

This study included patients with mild COVID-19 who did not require hospitalization. The total study duration per patient was 28 days including a screening period of two days, a randomization and treatment period of five days, and follow-ups on days five, 10, 14, and 28. Follow-up was either on-site or telephonically (subsequent visits after negative SARS-CoV-2 RT-PCR test).

Screening assessments included demographic information, medical history, current illness, laboratory results, physical examination, and vital signs. Local laboratories of the clinical sites performed the SARS-CoV-2 RT-PCR test in nasopharyngeal and/or oropharyngeal swabs based on the RdRp/N Gene. Cycle threshold (CT) value (from the RT-PCR test) is inversely proportional to the viral load and every ~3.3 increase in the CT value reflects a 10-fold reduction in viral load. CT values of 35 were considered negative.

During the scheduled follow-up visits, assessments included hospitalization status, clinical improvement based on the World Health Organization (WHO) 11-point Clinical Progression Scale [12], time to clinical improvement, SARS-CoV-2 RT-PCR status, physical examination, vitals, laboratory assessments, and presence of adverse events (AEs). AE severity was based on the CTCAE criteria version 5.0. All AEs that were life-threatening, fatal, required hospitalization, resulted in significant disability or incapacity, resulted in congenital anomalies, or were medically significant and required interventions to prevent any of the above were serious AEs (SAEs). Causality assessments were done by the study investigators based on the WHO-UMC Causality Assessment System [13].

Outcomes

The primary efficacy endpoint was the rate of hospitalization from randomization up to day 14. Hospitalization was defined as hospital admission for more than 24 hours with an RR of >24 breaths per minute and SpO_2_ ≤93% in room air, requiring oxygen supplementation. Secondary endpoints included the rate of hospitalization through day 28; the proportion of patients with clinical improvement (defined as a two-point decrease in the WHO clinical progression scale [12], rate of SARS-CoV-2 RT-PCR negativity, and change in SARS-CoV-2 viral load at the end of treatment (EOT)/day five, 10, and 14; and time to clinical improvement through day 14. Safety variables included the incidence and severity of treatment-emergent adverse events (TEAEs; clinical and laboratory) during the study and the proportion of patients who discontinued the study drug due to AEs.

Statistical analysis

A sample size of 1,218 patients is sufficient to detect a difference of 50% in hospitalization rates between molnupiravir and control groups by using Fisher’s exact test with a 10% expected hospitalization rate in the control group compared to 5% in the molnupiravir group, with 80% power at 5% level of significance and assumed drop-out rate of about 30%. A slightly higher dropout rate was assumed due to higher recovery rates in mild COVID-19 patients and apprehensions of recovered patients returning to hospitals for follow-up visits. A 50% reduction in the hospitalization rate was considered clinically relevant.

The safety population included all patients randomized who received at least one dose of the study medication. The modified intent-to-treat (mITT) population included all randomized patients with at least one post-baseline assessment. Per protocol population (PP) included all randomized patients who completed five days of treatment and 28 days of follow-up as per the protocol without any major protocol deviations. All efficacy analyses were performed on the mITT and PP populations except for hospitalization rate and mortality rate, which were analyzed only in the mITT population. The last observation was carried forward for secondary endpoint analysis in the mITT population. Safety analyses were performed on the safety population. All continuous demographic parameters were summarized using number, mean, median, standard deviation, and range. All categorical demographic parameters were summarized using numbers and percentages. Continuous variables were compared using a t-test, and the proportion of males/females was compared using Fisher’s exact test. The primary and secondary endpoints were summarized using descriptive statistics. The primary endpoint was analyzed using Fisher’s exact test. The secondary endpoints were analyzed using the chi-square test. The proportion of patients with clinical improvement was analyzed using the chi-square test, and time to clinical improvement was analyzed using survival analysis.

All statistical tests were performed at the p < 0.05 level of significance (two-sided) and presented with 95% confidence intervals. Adjustments for multiple testing were not performed. All statistical analysis was performed using the latest SAS® version system software (SAS Institute Inc., Cary, NC USA). AEs were coded using the latest version of the Medical Dictionary for Regulatory Activities (MedDRA version 24.0).

## Results

**Figure 1:**
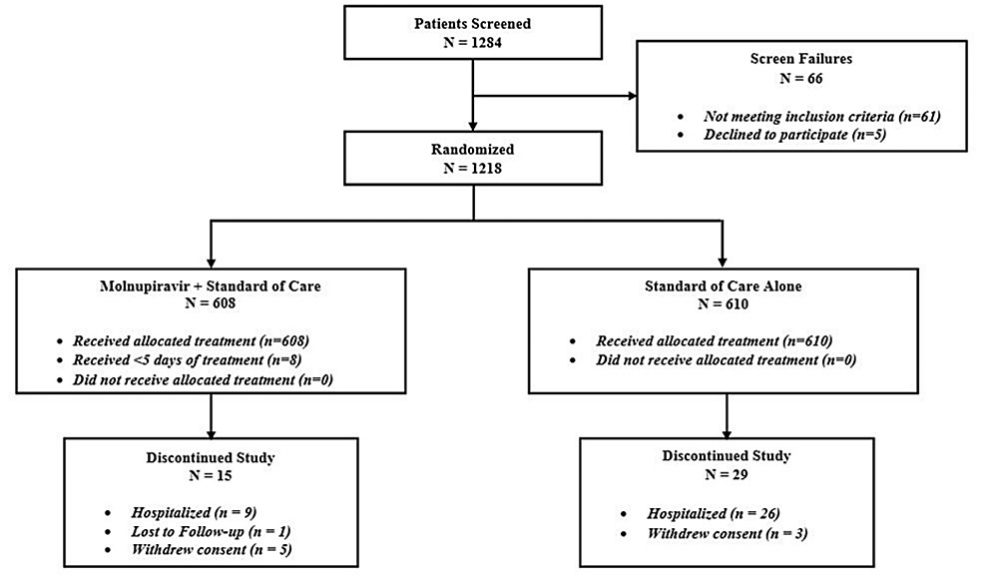
Patient disposition.

Of the 1,284 patients screened, 1,218 patients were randomized in 1:1 to molnupiravir (608 patients) and control (610 patients) groups. Of the 1,218 patients, 1,174 completed the study, and 44 were withdrawn. Reasons for withdrawal included disease progression (n = 35; 9/608 (1.48%) patients from the molnupiravir group and 26/610 (4.26%) patients from the control group), lost to follow-up (n = 1; 1/608 (0.16%) from the molnupiravir group), and withdrawal of consent to participate (n = 8; 5/608 (0.82%) patients from the molnupiravir group and 3/610 (0.49%) patients from the control group) (Figure 1).

The reasons for consent withdrawals were not provided by the patients. Out of the nine patients withdrawn from the study due to disease progression, 8/608 (1.31%) patients in the molnupiravir group did not complete five days of treatment. The primary endpoint (hospitalization rate) and mortality rate were analyzed in the mITT (n = 1,218) population and other secondary endpoints were analyzed in both the mITT and PP populations (n = 1,174). Demographic and baseline characteristics were similar between treatment groups (Table 1).

**Table 1:** Demography and other baseline characteristics. N = number of subjects in specified treatment; n = number of subjects in the specified category; SD = standard deviation; BMI = body mass index; SARS-CoV-2 = severe acute respiratory syndrome coronavirus 2; RT-PCR = reverse transcription polymerase chain reaction

Characteristics	Molnupiravir (N = 608), n (%)	Standard of care (N = 610), n (%)
Gender		
Male	408 (67.11)	425 (69.67)
Female	200 (32.89)	185 (30.33)
Race		
Indian	608 (100)	610 (100)
Age (years, mean ± SD)	35.2 ± 10.8	34.8 ± 10.8
Height (cm, mean ± SD)	165.6 ± 9.5	165.4 ± 9.4
Weight (kg, mean ± SD)	65.0 ± 9.1	64.2 ± 7.9
BMI (kg/m^2^, mean ± SD)	23.5 ± 2.6	23.4 ± 2.6
Comorbidities		
Obesity (BMI >30)	19 (3.12)	17 (2.78)
Diabetes mellitus	2 (0.32)	2 (0.32)
Hypertension	3 (0.49)	7 (1.14)
Time since symptom onset		
<3 days	327 (53.7)	335 (54.9)
3–5 days	281 (46.3)	275 (45.1)
SARS-CoV-2 RT-PCR test		
Cycle threshold value (mean ± SD)	25.9 (3.8)	25.9 (3.8)
Standard of care provided		
Multivitamins, antipyretics and antihistamines	478 (78.6)	472 (77.4)
Ivermectin	296 (48.68)	472 (77.38)
Inhalation budesonide	10 (1.6)	10 (1.6)

Efficacy

Overall, in the mITT population, the hospitalization rate from randomization to day 14 was 1.48% (9/608) in the molnupiravir group compared to 4.26% (26/610) in the control group for an absolute risk difference of 2.78% (95% CI = -4.65%, -0.90%, p = 0.0053) (Table 2). No further hospitalizations were reported in patients from day 14 to day 28.

**Table 2:** Comparison of the rate of hospitalization in the mITT population. ** P-values were obtained using the Fisher test. mITT = modified intent-to-treat

Parameter	Molnupiravir (N = 608), n (%)	Standard of care (N = 610), n (%)	Risk difference	95% confidence interval	Molnupiravir versus standard of care (p-values**)
Subjects hospitalized	9 (1.48)	26 (4.26)	-2.78	[-4.65, -0.9]	0.0053

In the mITT population, the proportion of patients with a two-point decrease on the WHO Clinical Progression Scale score (clinical improvement) in the molnupiravir group versus the control group was 80.76% versus 32.12% [-48.60 (-53.50, -43.80)], 95.56% versus 74.26% [21.30 (-25.10, -17.50)], and 97.37% versus 94.10% [3.30 (-5.50, -1.00)] at the end of day five, end of day 10, and end of day 14, respectively (Table 3).

**Table 3:** Proportion of patients with clinical improvement from baseline **P-values were obtained by using ANCOVA. mITT = modified intent-to-treat; PP = protocol population; EOT = end of treatment; ANCOVA = analysis of covariance

Visit	Molnupiravir (N = 608), n (%)	Standard of care (N = 610), n (%)	Proportional difference (95% confidence interval)	Molnupiravir versus standard of care (p-value**)	Molnupiravir (N = 593), n (%)	Standard of care (N = 581), n (%)	Proportional difference (95% confidence interval)	Molnupiravir versus standard of care (p-value**)
	mITT population				PP population			
Day 5 (EOT)	491 (80.76)	196 (32.13)	-48.6 (-53.5, -43.8)	<0.0001	489 (82.46)	194 (33.39)	-49.1 (-54.0, -44.2)	<0.0001
Day 10	581 (95.56)	453 (74.26)	-21.3 (-25.1, -17.5)	<0.0001	574 (96.80)	445 (76.59)	-20.4 (-24.1, -16.7)	<0.0001
Day 14	592 (97.37)	576 (94.10)	-3.3 (-5.5, -1.0)	0.0066	585 (98.65)	558 (96.04)	-2.6 (-4.4, -0.8)	0.0059

The median time to clinical improvement in the mITT population was 6.0 days (IQR = 6.00:7.00) versus 10 days (IQR = 6.00:11.00) in the molnupiravir group compared to the control group, showing statistically significant lesser time to clinical improvement (p < 0.0001) in the molnupiravir group. No deaths were reported in both groups for up to 28 days.

The RT-PCR negativity rates in nasopharyngeal and/or oropharyngeal swabs for SARS-CoV-2 in the molnupiravir group and control group in the mITT population were 77.14% versus 29.34% (p < 0.0001), 91.28% versus 70.16% (p < 0.0001), and 93.09% versus 89.02% (p = 0.0157) at the end of day five, at the end of day 10, and at the end of day 14, respectively.

**Figure 2:**
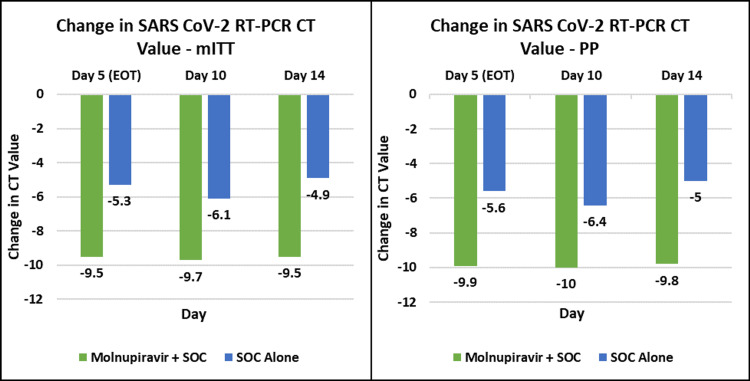
SARS-CoV-2 RT-PCR cycle threshold values over time. SARS-CoV-2 = severe acute respiratory syndrome coronavirus 2; RT-PCR = reverse transcription polymerase chain reaction; mITT = modified intent-to-treat; PP = protocol population; SOC = standard of care

The change in SARS-CoV-2 RT-PCR viral load (cycle threshold) in the mITT population from the baseline to the end of day five, end of day 10, and end of day 14 was 9.50 (5.20) versus 5.30 (4.90), 9.70 (6.40) versus 6.10 (7.70), and 9.50 (7.00) versus 4.90 (10.60), respectively, in the molnupiravir group compared to the control group (Figure 2) (Table 4).

**Table 4:** SARS-CoV-2 RT-PCR mean cycle threshold value change from baseline to day five, day 10, and day 14. SD = standard deviation; SARS-CoV-2 = severe acute respiratory syndrome coronavirus 2; RT-PCR = reverse transcription polymerase chain reaction; mITT = modified intent-to-treat; PP = protocol population; EOT = end of treatment

Visit	Molnupiravir (N = 608), mean (change from baseline ± SD)	Standard of care (N = 610), mean (change from baseline ± SD)	Molnupiravir versus standard of care (p-value**)	Molnupiravir (N = 593), mean (change from baseline ± SD)	Standard of care (N = 581), mean (change from baseline ± SD)	Molnupiravir versus standard of care (p-value**)
	mITT population			PP population		
Baseline	25.9 (3.8)	25.9 (3.8)		25.8 (3.8)	25.8 (3.9)	
Day 5 (EOT)	35.4 (9.5 ± 5.2)	31.2 (5.3 ± 4.9)	<0.0001	35.7 (9.9 ± 4.9)	31.4 (5.6 ± 4.8)	<0.0001
Day 10	35.5 (9.7 ± 6.4)	31.9 (6.1 ± 7.7)	<0.0001	35.8 (10.0 ± 6.2)	32.2 (6.4 ± 7.7)	<0.0001
Day 14	35.4 (9.5 ± 7.0)	30.7 (4.9 ± 10.6)	<0.0001	35.6 (9.8 ± 6.9)	30.8 (5.0 ± 10.9)	<0.0001

Safety

A total of 47 AEs were reported in 45 patients. In total, 30 AEs were reported in 29/608 (4.80%) patients in the molnupiravir group and 17 AEs in 16/610 (2.60%) patients in the control group (Table 5). Headache, nausea, diarrhea, and oropharyngeal pain were the most commonly reported AEs during the study period, with 7/30 (23.33%), 6/30 (20.00%), 4/30 (13.33%), and 2/30 (6.66%), AEs, respectively in the molnupiravir group. Of the 30 AEs reported in the molnupiravir group, 13/30 (43.33%) AEs were considered to be related to the study medication (Table 5). All reported AEs were mild in severity and resolved completely. No deaths or SAEs were reported in the study. No clinically significant changes in vital signs or laboratory findings were recorded, and there were no other observations related to the study drug’s safety in this study. No patients discontinued the study due to AEs.

**Table 5:** Treatment-emergent adverse events with relatedness-safety population. Each patient counted once per preferred term and once per system organ class. E = number of treatment-emergent adverse events

System organ class preferred term	Test (N = 608)		Reference (N = 610)		Overall (N = 1,218)	
	Related, n (%) E	Not related, n (%) E	Related, n (%) E	Not related, n (%) E	Related, n (%) E	Not related, n (%) E
Any treatment-emergent adverse event	13 (2.1) 13	16 (2.6) 17	5 (0.8) 6	11 (1.8) 11	18 (1.5) 19	27 (2.2) 28
Gastrointestinal disorders	7 (1.2) 7	7 (1.2) 7	0 (0)	3 (0.5) 3	7 (0.6) 7	10 (0.8) 10
Abdominal pain	0 (0)	0 (0)	0 (0)	1 (0.2) 1	0 0	1 (0.1) 1
Abdominal pain upper	1 (0.2) 1	0 (0)	0 (0)	0 0	1 (0.1) 1	0 0
Diarrhea	0 (0)	4(0.7) 4	0 (0)	1 (0.2) 1	0 (0)	5 (0.4) 5
Hyperchlorhydria	0 (0)	2 (0.3) 2	0 (0)	0 (0)	0 (0)	2 (0.2) 2
Nausea	6 (1.0) 6	0 (0)	0 (0)	0 (0)	6 (0.5) 6	0 0
Vomiting	0 (0)	1 (0.2) 1	0 (0)	1 (0.2) 1	0 (0)	2 (0.2) 2
General disorders and administration site conditions	0 (0)	3 (0.5) 3	0 (0)	0 (0)	0 (0)	3 (0.2) 3
Asthenia	0 (0)	1 (0.2) 1	0 (0)	0 (0)	0 (0)	1 (0.1) 1
Fatigue	0 (0)	1 (0.2) 1	0 (0)	0 (0)	0 (0)	1 (0.1) 1
Swelling	0 (0)	1 (0.2) 1	0 (0)	0 (0)	0 (0)	1 (0.1) 1
Metabolism and nutrition disorders	0 (0)	0 (0)	0 (0)	1 (0.2) 1	0 (0)	1 (0.1) 1
Decreased appetite	0 (0)	0 (0)	0 (0)	1 (0.2) 1	0 (0)	1 (0.1) 1
Musculoskeletal and connective tissue disorders	0 (0)	0 (0)	0 (0)	1 (0.2) 1	0 (0)	1 (0.1) 1
Pain in extremity	0 (0)	0 (0)	0 (0)	1 (0.2) 1	0 (0)	1 (0.1) 1
Nervous system disorders	4 (0.7) 4	4 (0.7) 4	4 (0.7) 5	6 (1.0) 6	8 (0.7) 9	10 (0.8) 10
Headache	3 (0.5) 3	4 (0.7) 4	2 (0.3) 2	5 (0.8) 5	5 (0.4) 5	9 (0.7) 9
Somnolence	1 (0.2) 1	0 0	3 (0.5) 3	1 (0.2) 1	4 (0.3) 4	1 (0.1) 1
Respiratory, thoracic, and mediastinal disorders	2 (0.3) 2	1 (0.2) 1	1 (0.2) 1	0 (0)	3 (0.2) 3	1 (0.1) 1
Hiccups	0 (0)	1 (0.2) 1	0 0	0 (0)	0 0	1 (0.1) 1
Oropharyngeal pain	2 (0.3) 2	0 0	1 (0.2) 1	0 (0)	3 (0.2) 3	0 0
Skin and subcutaneous tissue disorders	0 (0)	2 (0.3) 2	0 (0)	0 (0)	0 (0)	2 (0.2) 2
Rash	0 (0)	2 (0.3) 2	0 (0)	0 (0)	0 (0)	2 (0.2) 2

## Discussion

There is a great need for an effective oral antiviral drug to fight against the COVID-19 pandemic, which can be easily administered on an outpatient basis. Few oral antiviral drugs (i.e., Favipiravir and Umifenovir) were approved for mild-to-moderate COVID-19 without clear evidence of virological clearance and prevention of progression from mild-to-moderate/severe disease. While therapies such as remdesivir and monoclonal antibodies are available, these cannot be given orally, making them less suitable for outpatient management.

Molnupiravir (MK4482/EIDD2801) is a potent ribonucleoside analog that inhibits the replication of multiple RNA viruses including SARS-CoV-2, which has shown in preclinical studies to be a promising antiviral agent for breaking the chains of transmission of SARS-CoV-2 in the population. Cox et al. [14] reported that the infected animals treated with MK4482/EIDD2801 twice daily significantly reduced the viral load of SARS-CoV-2 in the upper airways and completely suppressed the spread to untreated contact animals. Fischer et al. [15] in a phase 2a trial reported virus isolation was 0% versus 11.1% (p = 0.03) on day five, p = 0.01 for viral RNA clearance, SARS-CoV-2 negativity was 92.5% versus 80.3%, by day 28 antibodies to SARS-CoV-2 was 99.2% versus 96.5% under 800 mg molnupiravir versus placebo.

In this randomized, open-label, phase III, comparative clinical trial of 1,218 patients with mild COVID-19, participants receiving molnupiravir 800 mg twice daily for five days showed statistically signiﬁcant fewer hospital admissions, faster time to clinical improvement, early SARS-CoV-2 RT-PCR negativity rates in comparison to those who received standard of care treatment alone. Compared to the control group, the molnupiravir group has also shown early and statistically significant decreases in viral load (CT value) from baseline. The incidence of AEs was comparatively higher in the molnupiravir group (4.80%) than in the control group (2.60%). There were no deaths reported in either group, all AEs were non-serious, were of mild severity, and none resulted in drug discontinuation. The most commonly reported AEs were nausea, diarrhea, and headache, which were completely resolved. The safety profile of the molnupiravir arm in this study was consistent with the data reported in the literature from clinical studies. In a similar Merck phase 3 study in mild-to-moderate COVID-19, the most common adverse reactions (≥1% of subjects) reported during treatment were diarrhea (3%), nausea (2%), dizziness (1%), and headache (1%), all of which were Grade 1 (mild) or Grade 2 (moderate) [16]. In their phase 3 study, Merck reported 6.80% versus 9.70% (risk difference = 2.90%; 95% CI = 0.1; 5.9; p = 0.02) hospitalization or death rate from randomization to day 29 in the molnupiravir group compared to the placebo group [16]. Because the current study was not placebo-controlled, a direct correlation of these results with the above-mentioned literature is not possible, but the treatment outcomes of the molnupiravir group can be compared.

This study clearly showed the efficacy and safety of oral molnupiravir among mild COVID-19 outpatients. Because of its oral route of administration, it is suitable and convenient for outpatient administration.

Limitations

Limitations of this study included the open-label study design to allow for the treatment complexities due to SoC treatments. This might account for more patients receiving ivermectin in the control group. However, central randomization ensured treatment allocation was done without the involvement of physicians/patients. Hospitalization was clearly defined based on objective parameters (SpO_2_, RR, and duration of hospitalization), ensuring the elimination of assessment bias in this open-label design.

The rapid rate of disease progression and the emergent consequences did not allow for a placebo-controlled design. Only patients with mild COVID-19 were enrolled. Though inclusion criteria did not necessitate patients to be at risk for disease progression, patients with comorbid conditions such as obesity (body mass index ≥30 kg/m^2^), diabetes mellitus, and hypertension were not excluded either. This trial did not actively collect information about comorbidities, but only collected self-reported data from patients, which could be an explanation for the lower reporting rates of comorbidities than would be expected in the general Indian population.

## Conclusions

Oral antivirals are required to treat for the prevention and progression of COVID-19 to severe illness and transmission of SARS-CoV-2. Molnupiravir is the first oral, direct-acting antiviral that has shown a favorable safety and tolerability profile and has been demonstrated to be highly effective at reducing nasopharyngeal SARS-CoV-2 infectious virus and viral RNA in preclinical and clinical studies. The results of our study also demonstrated that molnupiravir was effective, safe, and well tolerated in mild COVID-19, with a reduction in hospitalization rates, faster time to clinical improvement, and SARS-CoV-2 RT-PCR negativity. Molnupiravir can play a major role in the treatment of SARS-CoV-2 infection and can prevent the progression to moderate/severe disease, thus reducing/eliminating the transmission of SARS-CoV-2. Additionally, shortening the infection phase can reduce the emotional and socioeconomic costs of prolonged patient isolation.
